# Potential Utility of Systemic Plasma Biomarkers for Evaluation of Pediatric Schistosomiasis in Western Kenya

**DOI:** 10.3389/fimmu.2022.887213

**Published:** 2022-05-06

**Authors:** Bartholomew N. Ondigo, Rachael E. Hamilton, Edwin O. Magomere, Isaac O. Onkanga, Pauline N. Mwinzi, Maurice R. Odiere, Lisa Ganley-Leal

**Affiliations:** ^1^ Department of Biochemistry and Molecular Biology, Faculty of Science, Egerton University, Egerton, Kenya; ^2^ School of Public Health, Boston University, Boston, MA, United States; ^3^ Global Development, Elegance Biotechnologies, Wayne, PA, United States; ^4^ Centre for Global Health Research, Kenya Medical Research Institute (KEMRI), Kisumu, Kenya; ^5^ Regional Office for Africa, World Health Organization, Brazzaville, Democratic Republic of Congo

**Keywords:** biomarkers, schistosomiasis, infection, intensity, diagnosis

## Abstract

**Introduction:**

Current diagnostic tools for schistosomiasis are limited, and new tests are necessary to enhance disease diagnosis and surveillance. Identification of novel disease-specific biomarkers may facilitate the development of such tests. We evaluated a panel of biomarkers used in sepsis and parasitic diseases for their potential suitability in the diagnosis of schistosomiasis.

**Objective:**

The study evaluated the levels of systemic plasma biomarkers in relation to *Schistosoma mansoni* infection and parasite burden.

**Methods:**

Six biomarkers were measured in the plasma of children from schistosomiasis-endemic regions using ELISA. The concentration of soluble CD23 (sCD23) and lipopolysaccharide (LPS) was tested in 199 and 124 plasma samples, respectively, while interleukin-6 (IL-6), soluble triggering receptor expressed on myeloid (sTREM) cells, eotaxin-1, and fatty acid-binding protein (FABP) concentrations were tested in 30 plasma samples.

**Results:**

The concentration of IL-6, eotaxin-1, FABP, and LPS was similar between schistosome-infected and uninfected children. The schistosome-infected children had higher median levels of sTREM and sCD23 as compared to uninfected children, 119.0 (29.9–208.9) versus 10.7 (0.0–73.4) (*p* = 0.046) and 2,549.0 (1,899.0–3,356.0) vs. 2,035.0 (1,448.0–2,939.0) (*p* = 0.05), respectively. In addition, sTREM was positively correlated with egg density (*p* = 0.017).

**Conclusion:**

Our data show that active schistosomiasis *per se* is associated with elevated levels of sTREM and sCD23. sTREM has potential diagnostic and prognostic values. However, these biomarkers did not distinguish between children with low egg burden and uninfected children.

## Introduction

Schistosomiasis is a tropical disease affecting communities with limited access to safe water and with inadequate sanitation (1, 2). The disease affects over 200 million people worldwide, about 90% of whom reside in Sub-Saharan Africa. A significant number (123.6 million) of those affected are children (3). Children from endemic areas are infected at the age of 2 years and may remain chronically infected throughout their school-going age (4). The disease results in 3.3 million disability-adjusted life years (DALYs) lost annually due to overt and sub-clinical morbidities (5). In Kenya, about 17.4 million people are at risk of schistosomiasis (6, 7). The main parasite species causing infections in Kenya are *Schistosoma haematobium* and *Schistosoma mansoni* (6, 8, 9).

Immune molecules such as interleukin-6 (IL-6), soluble triggering receptor expressed on myeloid (sTREM), eotaxin-1, fatty acid-binding protein (FABP), soluble CD23 (sCD23), and lipopolysaccharide (LPS) have been evaluated in previous studies as potential diagnostic markers for parasitic infections and sepsis (10–13). Triggering receptor expressed on myeloid cells-1 (TREM-1) is a transmembrane receptor expressed by innate immune cells, including endothelial cells, mature monocytes, and macrophages and platelets (14). In addition to its expression in a cell membrane-bound form, TREM-1 is released as a soluble factor (sTREM-1). sTREM-1 has been investigated and is a reliable biomarker of disease severity and outcome in septic shock (15). TREM-1 is increased in the skin, biological fluids, and tissues with bacterial and fungal infections (16, 17). sTREM-1 released into the blood and other bodily fluids interacts with a 12-kDa DNAX-activating protein (DAP12) amplifying pathogen-induced signals (18). This interaction triggers the release of pro-inflammatory cytokines including IL-1b and IL-8 and monocyte chemotactic protein (19). A gradual increase in levels of sTREM has been observed in *S. mansoni* over the course of infection (20), which appeared to upregulate *DAP12* and *IL-8* gene expression, suggesting the important role of sTREM in parasitic infections (21).

IL-6 and eotaxin-1 levels are altered among individuals with parasitic infection (22), suggesting their potential utility in diagnosis (22). FABP induces protective immunity against *S. mansoni* infection by triggering a Th1-like immune response during infection (23); as such, its plasma concentration can be monitored to assess disease progression. CD23 is a surface membrane receptor for IgE on B cells. The receptor is initially expressed as a 45-kDa type II membrane protein and subsequently released as sCD23 fragments by the action of an endogenous metalloprotease (24). sCD23 levels were increased with schistosome infection intensity but declined significantly with schistosome-specific IgE levels (12). Elevated levels of serum sCD23 prior to diagnosis were associated with an increased risk of non-Hodgkin lymphoma (25). Increased synthesis of CD23 signals a corresponding increase in the synthesis of its ligand (IgE) (24, 26). CD23 expressed on the B-cell surface may bind IgE and regulate production and concentration in plasma (19). A previous study demonstrated the role of sCD23 in developing resistance to reinfection in schistosomiasis (27). Nonetheless, elevated levels of LPS in plasma of hepatosplenic schistosomiasis caused by *S. mansoni* have been demonstrated to be potential biomarkers for diagnosis of schistosomiasis (28).

The Kato–Katz technique is the gold standard method for the diagnosis of schistosomiasis. This tool relies on the detection of eggs in stool for most species apart from *S. haematobium*, whose eggs are detected in urine (29). A major setback of using the microscopy method is that it has a low sensitivity among individuals with low parasite burden and its dependence on the appearance of eggs in the stool, which may occur 6–8 weeks after infection (30). To improve the sensitivity and accuracy of diagnosis, an opportunity exists for developing biomarker-based diagnostic tools that can complement microscopy and help to monitor disease progression. We sought to investigate whether systemic plasma biomarkers can be used to distinguish between children infected with schistosomiasis and healthy controls (uninfected children). We also assessed the correlation between the biomarkers and their ability to distinguish infection intensities (light, moderate, and heavy) and changes in hemoglobin levels. The study hypothesized that children infected with *S. mansoni* would have unique biomarker profiles that could ultimately be exploited to aid in diagnosis.

The diagnosis of schistosomiasis currently relies on microscopic detection of schistosome eggs in stool or urine samples and serological assays. Identification of schistosomiasis-specific biomarkers will complement existing diagnostic methods with the added advantage of early diagnosis before schistosome eggs appear in stool and the ability to track disease progression. The optimization of novel diagnostic approaches may be accomplished by the selection of specific biomarkers of infection. In this study, we assessed the potential utility of systemic plasma biomarkers for the evaluation of pediatric schistosomiasis in western Kenya.

## Materials and Methods

### Study Population

Samples analyzed in this study were collected from children aged between 10 and 12 years from Lwanda Kotieno in Uyoma, Rarieda District, a schistosomiasis-endemic region in western Kenya. Eligible children provided stool samples for three consecutive days. The stool samples were used to diagnose the presence of *S. mansoni* eggs based on the Kato–Katz technique. At the enrolment visit, the study nurse conducted a clinical examination consisting of an assessment of nutritional status, body temperature, determination of liver or spleen sizes (by measuring extensions below the rib cage), and palpation of liver and spleen [for determination of the firmness of the organ(s)]. Height and weight were also measured and used to calculate a body mass index (BMI). Mid-upper arm circumference (MUAC) was measured to assess nutritional status. All plasma samples were from archived specimens collected as part of an ongoing study. Thus, all samples were collected from well-characterized participants. A total of 199 and 124 samples were analyzed for sCD23 and LPS, respectively. A subset of samples was assayed for IL-6, sTREM, eotaxin-1, and FABP. The uninfected control children were those attending the same schools but with stools samples negative for *S. mansoni* eggs at three continuous time points. The sample sizes tested were chosen based solely on the sufficiency of plasma volume to allow for the testing of multiple analytes.

### Ethical Approval

This study was approved by the Scientific and Ethics Review Unit (SERU) of Kenya Medical Research Institute (KEMRI) (protocol # SERU/KEMRI/CGHR/009/3025). Written parental informed consent and child assent were obtained for all participants.

### Sample Collection and Storage

Blood samples were collected in heparinized tubes and transported on ice to KEMRI-CGHR, Neglected Tropical Diseases (NTD) laboratory in Kisumu within 6 h of collection. In the laboratory, a blood sample was fractioned by centrifuging for 10 min at approximately 2,000 × *g*. Plasma was then aspirated and stored in a 0.5-ml Sarstedt tube at −20°C until further analysis. All the samples had never been thawed prior to the assays in this study. Stool samples for the detection of eggs were collected in sterile aluminum bags. The bags were issued to each participant labeled with a unique identifier number. An oral description of the use and proper handling of the stool bag and samples was given, and each participant was instructed to collect a fresh stool sample. Upon reception, samples were assessed for possible contamination and volume adequacy. The samples were transported in a cooler box within 6 h of collection to KEMRI-CGHR, NTD Laboratory, in Kisumu for analyses.

### Stool Sample Microscopy

Stool samples were processed using the Kato–Katz technique for the detection of *S. mansoni* eggs based on duplicate slides using the 41.7-mg template (31). Slides were viewed under a light microscope by two independent microscopists under ×40 magnification. The eggs counted for each sample were recorded as eggs per gram (EPG), while samples with zero eggs were recorded as negative. The number of *S. mansoni* eggs was counted, recorded, and multiplied by 24 to determine the number of eggs per gram of feces. Infection intensity was classified as light (1–99 EPG), medium (100–399 EPG), or heavy (≥400 EPG) according to the WHO guidelines.

### Assessment of Interleukin-6, Soluble Triggering Receptor Expressed On Myeloid, Eotaxin-1, and Fatty Acid Binding Protein

Plasma levels of IL-6, sTREM, eotaxin-1, and FABP were measured by commercially available quantikine ELISAs (R&D Systems, Minneapolis, MN, USA) according to the manufacturer’s protocol. Briefly, 100 µl in duplicate of standards, controls, or plasma samples was transferred to appropriate wells; the plate was then covered with an adhesive seal and incubated for 1 h at room temperature (RT) to allow for the target proteins to bind plate-coated antibodies. The unbound proteins were washed four times using the wash buffer in a plate washer. One hundred microliters of diluted biotinylated antibodies was added to each well and allowed to bind to captured biomarkers for 1 h at RT. At the end of the incubation period, six rounds of wash were performed, and 100 µl of the diluted streptavidin–peroxidase conjugate was added and incubated for 1 h at RT. Detection was performed by adding tetramethylbenzidine (TMB) substrate to the washed plate, and the reaction was allowed to take place for 20 min. The reaction was stopped by adding 100 µl of stop solution, and the plates were immediately read at 450 nm on a Spectramax Emax plate reader (Molecular Devices, San Jose, CA, USA). Absorbance was recorded for each biomarker and converted to concentration using the standard curve.

### Assessment of sCD23

The sCD23 ELISA (catalog # BMS 227-2) was performed according to the manufacturer’s recommendation (Bender MedSystems, Vienna, Austria).

### Assessment of Plasma Lipopolysaccharide

Plasma levels were evaluated using the chromogenic endpoint LPS amoebocyte lysate detection assay (catalog # A39553, ThermoFisher Scientific, Waltham, MA, USA) according to the manufacturer’s instructions. Samples were measured on a microplate absorbance reader at 405 nm on a Spectramax Emax plate reader (Molecular Devices, San Jose, CA, USA). The kit had a minimum detection limit of 0.01 EU/ml.

### Statistical Analysis

Medians were used as measures of central tendency. Comparisons between two groups were performed using the Mann–Whitney U test for non-normally distributed variables. The comparison between three groups was evaluated using the Kruskal–Wallis test, and the strength of association between variables was analyzed using a Spearman’s rank-order correlation (r_s_). Data were considered statistically significant at *p* ≤ 0.05. All statistical analyses were performed using GraphPad Prism version 6.0 for Windows (GraphPad Software, Inc., San Diego, CA, USA).

## Results

A total of 199 children comprised the final dataset for analysis. The children’s ages, other general characteristics, and the status of their parasitological infections are presented in **Table 1**.

**Table 1:** Demographic characteristics of the study participants and the status of infection.

Demographic characteristic	Infected participants (n = 177)	Healthy uninfected controls (n = 22)
Median (IQR) years	11 (10–11)	11 (10–12)
Sex (no. M/no. F)	97/80	8/14
Median (IQR) BMI	15.36 (14–16.59)	15.89 (14.61–17.40)
Median (IQR) MUAC	18.45 (17.4–19.5)	18.86 (18.15–19.96)

n, count; m, male; f, female; IQR, interquartile range; BMI, body mass index; MUAC, mid-upper arm circumference.

### Concentration of Biomarkers by Infection Status: Soluble Triggering Receptor Expressed On Myeloid Is Elevated in Children With Schistosomiasis

We compared the levels of biomarkers (IL-6, sTREM, eotaxin-1, FABP, sCD23, and LPS) between children with schistosomiasis (detectable eggs) and uninfected children (undetectable eggs). There was a significant difference in concentration of sTREM and sCD23 between the infected and uninfected children (*p* = 0.046 and *p* = 0.05, respectively). The median levels of plasma IL-6, eotaxin-1, FABP, and LPS were marginally higher in the infected group but not different from the uninfected group (**Table 2**).

**Table 2:** Comparison of median serum level of biomarkers between infected and uninfected children.

Median level (25th–75th percentile)			
Biomarkers	Infected individuals n = 177	Healthy controls n = 22	*p*
IL-6	0.87 (0.2–3.9)	0.39 (0.0–4.8)	0.594
sTREM	119.00 (29.9–208.9)	10.65 (0.0–73.4)	0.046*
Eotaxin-1	30.15 (11.0–49.0)	15.59 (10.8–23.3)	0.144
FABP	315.01 (174.7–507.2)	419.06 (382.3–559.2)	0.201
sCD23	2,548.60 (1,899.0–3,356.0)	2,035.09 (1,448.0–2,939.0)	0.050*
LPS	0.26 (0.1–0.5)	0.18 (0.0–0.5)	0.773

The analysis was performed with Mann–Whitney U.

IL-6, interleukin-6; sTREM, soluble triggering receptor expressed on myeloid; FABP, fatty acid-binding protein; sCD23, soluble CD23; LPS, lipopolysaccharide.

^*^Significant difference at p ≤ 0.05.

### Concentration of Biomarkers by Infection Intensity: Soluble Triggering Receptor Expressed on Myeloid Discriminates Between Light, Moderate, and Heavy Infection

**Figure 1:**
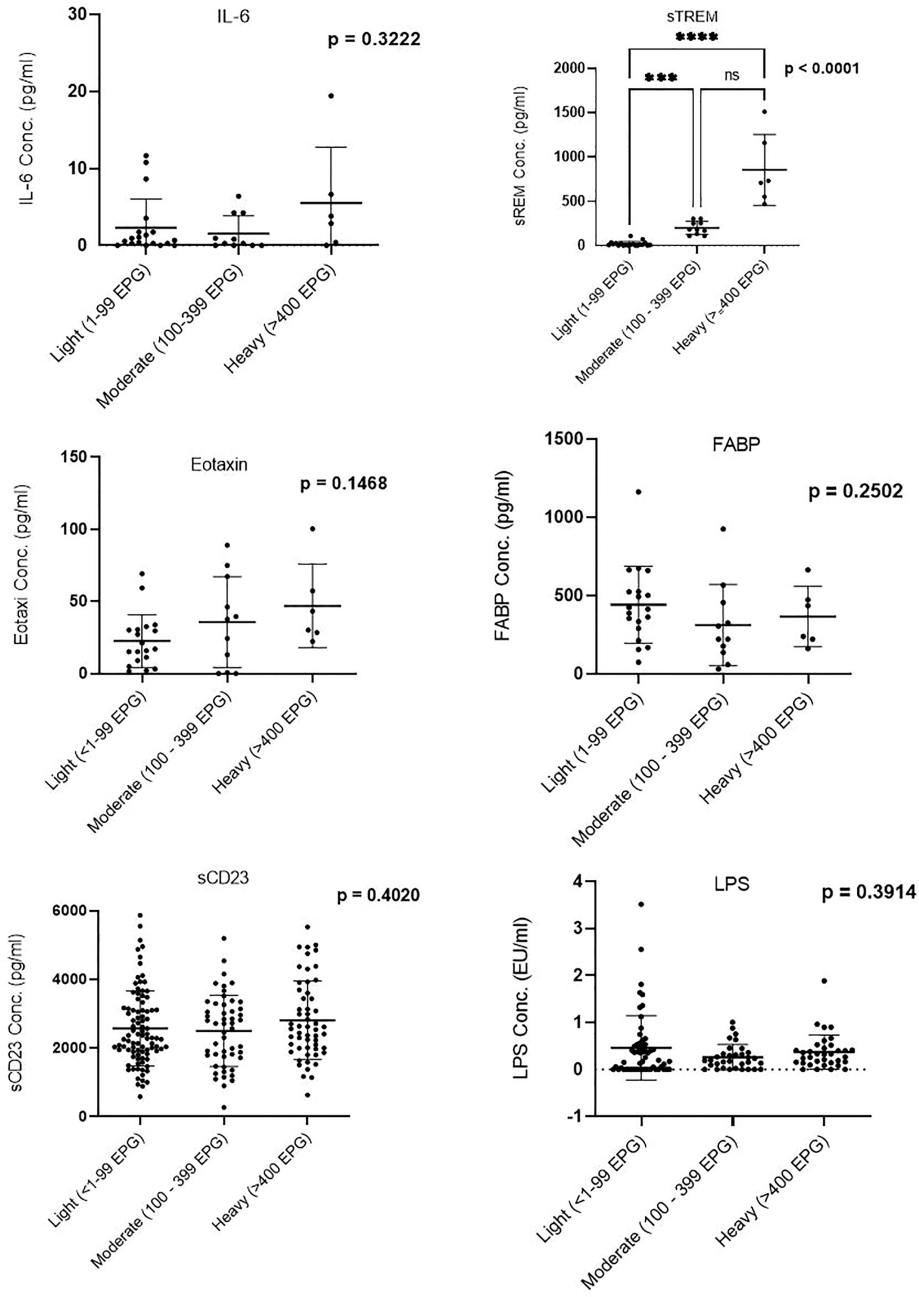
Comparison of biomarker plasma concentration in infection groups. Groups were categorized based on the egg counts into low (1–99 EPG), moderate (100–399 EPG), and heavy (≥400 EPG). EPG, eggs per gram. *** (Moderatly significant, p < 0.0008); **** (Highly significant level p<00001). ns, not significant.

Plasma concentration of sTREM showed a difference between the three infection groups: light, moderate, and heavy; *p* < 0.0001. Between-group comparisons of other biomarkers (IL-6, eotaxin-1, FABP, sCD23, and LPS) did not show significant differences (**Figure 1**).

### Biomarker Correlation: Soluble Triggering Receptor Expressed on Myeloid Correlates With Eotaxin-1 and Interleukin-6 But Not With Eggs per Gram

**Figure 2:**
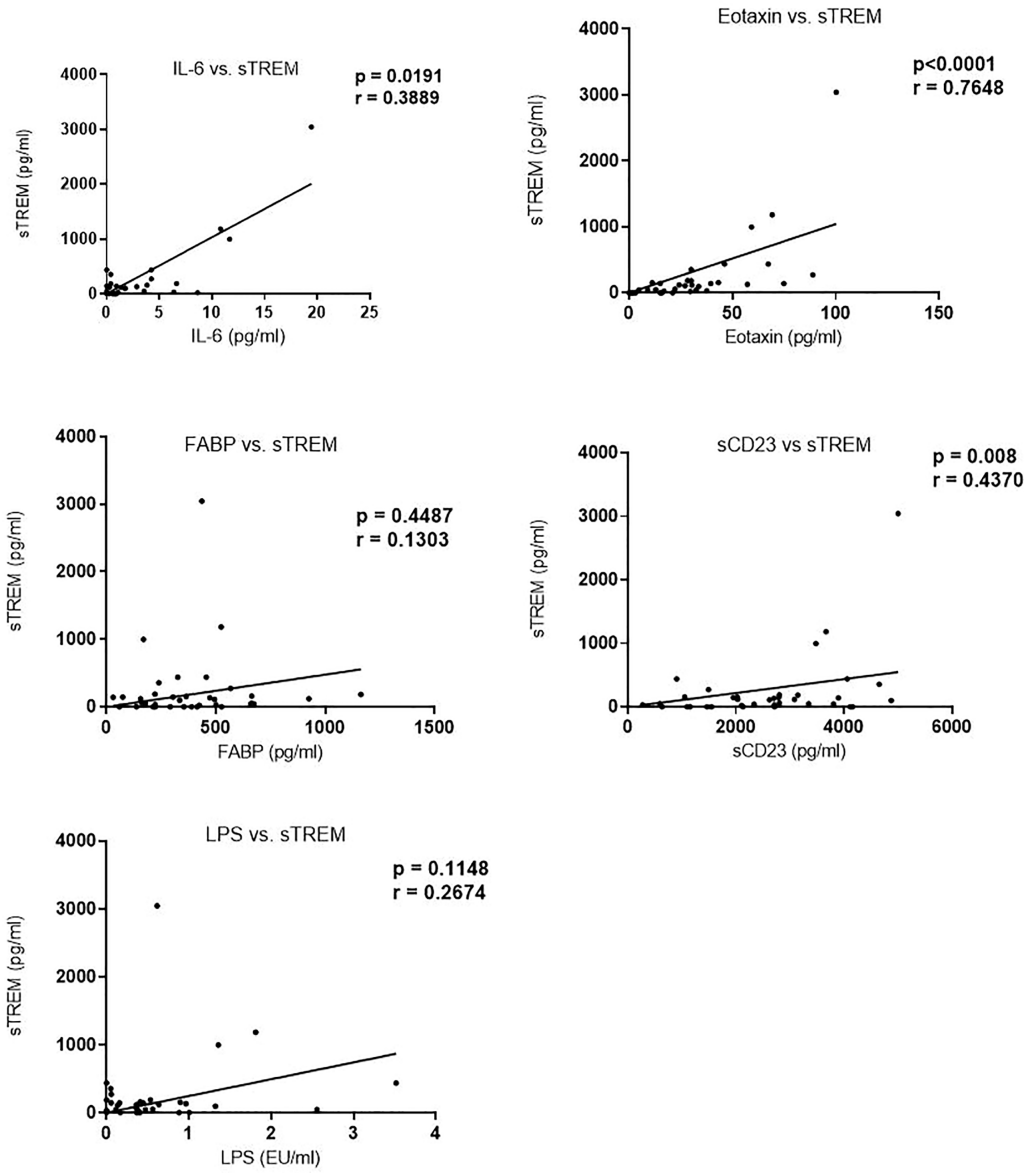
Correlation between sTREM and other biomarkers in children in western Kenya. Data plotted are those with corresponding values (n = 36) for each of the biomarkers. sTREM, soluble triggering receptor expressed on myeloid.

**Figure 3:**
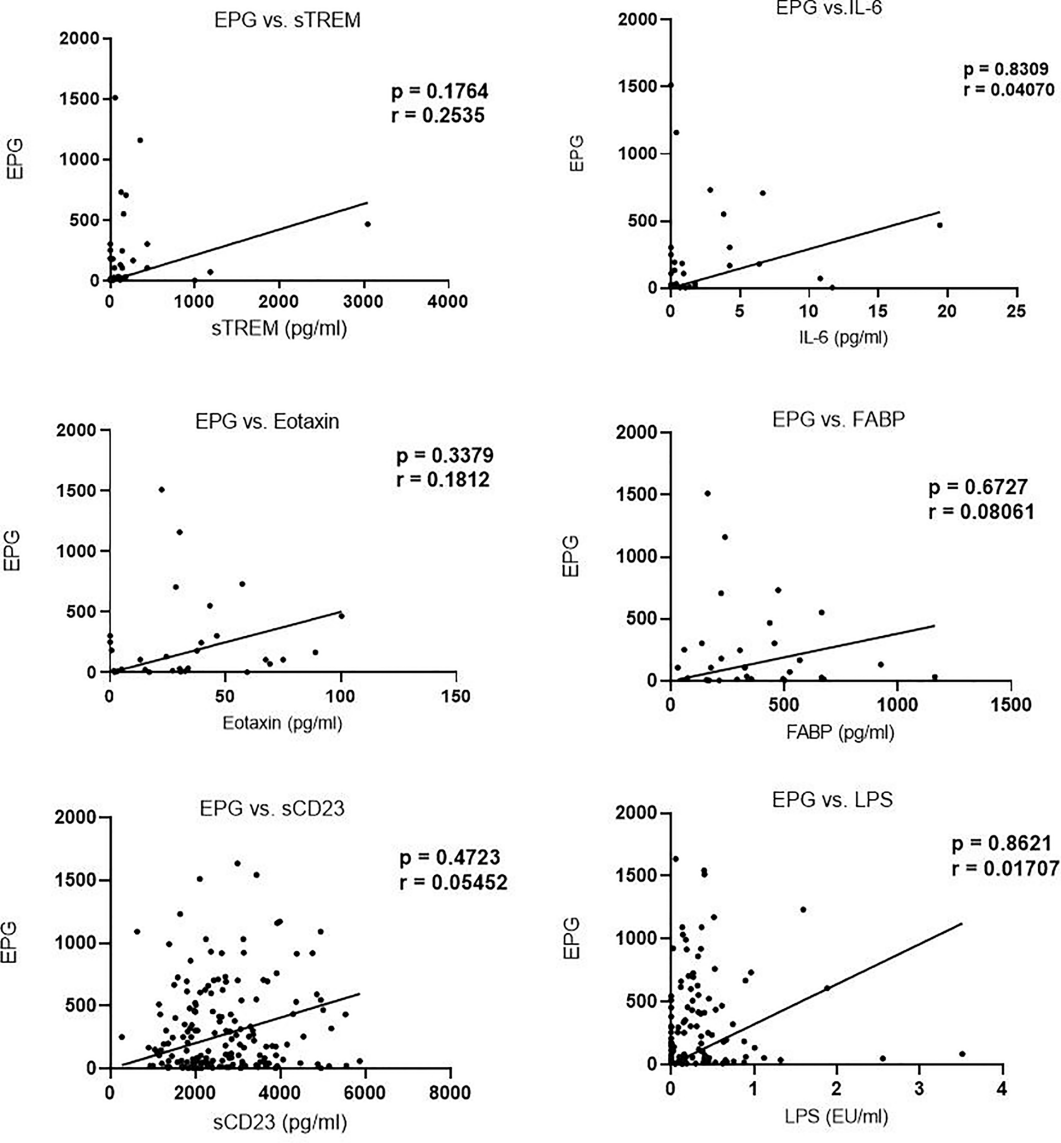
Correlation between biomarker concentration and eggs per gram (EPG) among infected children.

We next evaluated the correlation of sTREM with other biomarkers. There was a strong correlation between sTREM and eotaxin-1 (*p* < 0.0001, r = 0.7628), sTREM and IL-6 (*p* = 0.0191, r = 0.3898), and sTREM and sCD23 (*p* = 0.008, r = 0.4370). LPS and FABP did not correlate significantly with sTREM (**Figure 2**). Evaluation of the association of various biomarkers with EPG revealed no significant correlations (**Figure 3**), suggesting that presence of eggs in stool did not affect biomarker concentration.

### Correlation of Biomarkers With Hemoglobin Levels: Biomarker Concentration Is Not Associated With Hemoglobin Levels

**Figure 4:**
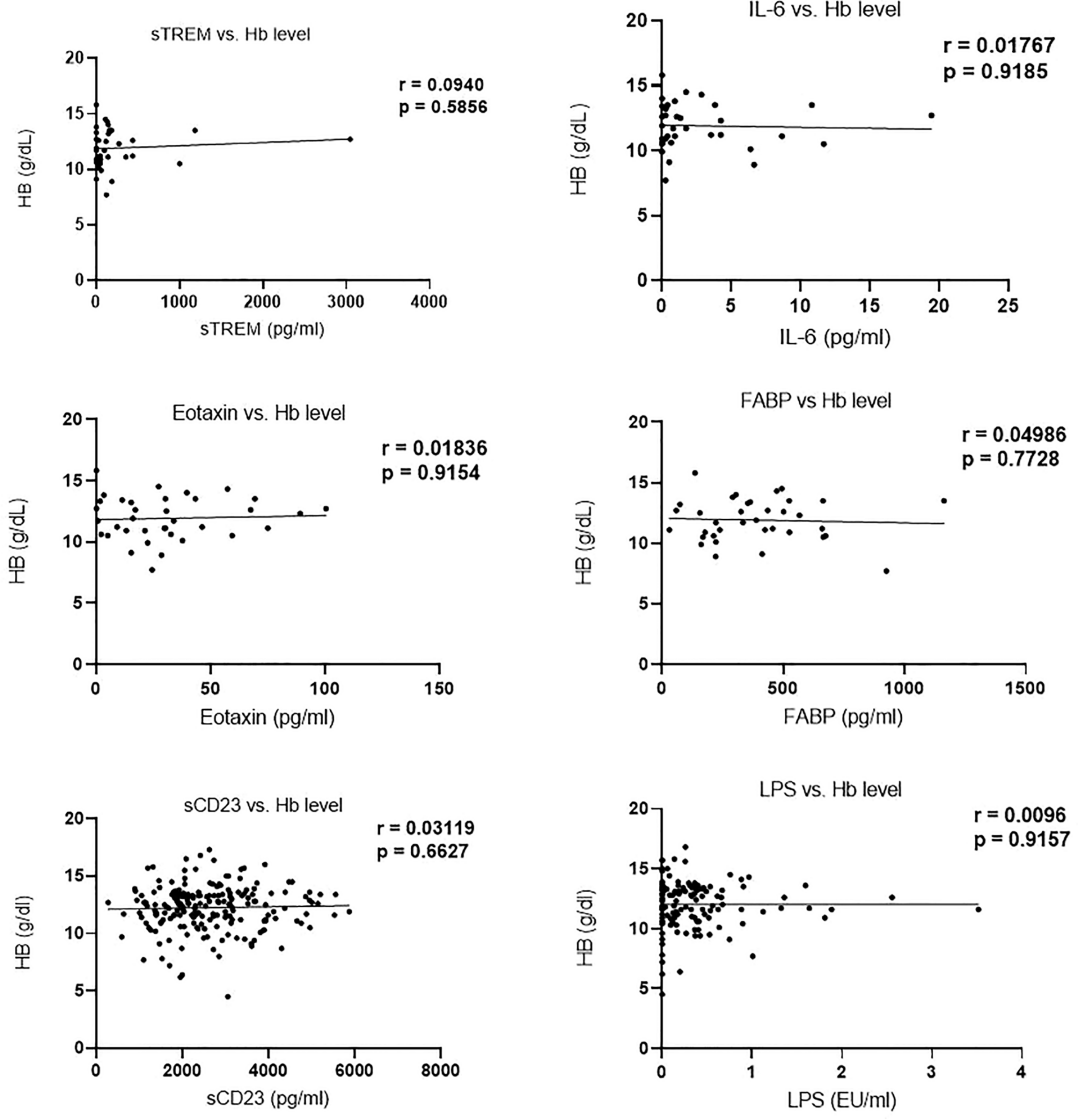
Correlation of biomarker concentration and hemoglobin (g/dl) levels among study participants.

Analysis of the correlation between biomarker (sTREM, IL-6, eotaxin-1, FABP, sCD23, and LPS) concentration and hemoglobin levels revealed no significant correlation. Moreover, Hb levels in egg positive and egg negative children were not different (**Figure 4**).

## Discussion

Human schistosome parasites can be eliminated from the body, and the disease can be cured before progressing to complications if early-stage and sensitive diagnostic approaches are available. Additionally, prompt treatment can reverse morbidity (32). However, the currently used gold standard method is not sensitive and can only detect *Schistosoma* eggs in stool 6–8 weeks after infection (33). Therefore, there is a need to develop robust tools for the diagnosis of human schistosomiasis. In this study, the utility of systemic biomarkers (IL-6, sTREM, eotaxin-1, FABP, sCD23, and LPS) was assessed to determine their potential use as diagnostic markers.

This study reported significantly higher levels of sTREM in infected compared to uninfected children. The level of sTREM also increased with egg burden, discriminating between light, moderate, and heavy infections, *p* < 0.001. A subtle increase in levels of IL-6, eotaxin-1, FABP, sCD23, and LPS among schistosomiasis-infected children signaled a change in the concentration of these systemic biomarkers during schistosomiasis triggered by an immune response. Increased secretion of cytokines and chemokines such as IL-6 and eotaxin-1 (CCL11) during schistosomiasis infection has been reported (34). IL-6 has both pro-inflammatory and anti-inflammatory activities (35), while chemokine eotaxin-1 plays a role in parasite-induced inflammation by mediating mobilization, recruitment, and proliferation of primary eosinophils to the site of infection (22). Their participation in immune response warrants observed increased concentration among *S. mansoni* egg-positive children, suggestive of an increase in synthesis and secretion of these systemic biomarkers. The increased secretion of sTREM among schistosomiasis-infected children is noteworthy considering the primary role in amplifying the inflammatory response to parasite infection through the adaptor protein DAP12 (18, 36). Moreover, sTREM mediates macrophage activation in response to microbial infection, and increased levels have been demonstrated in schistosome infections (20).

FABP constitutes a family of transport proteins that have been shown to induce protective immunity against *S. mansoni* (23). However, despite its role in schistosomiasis, its correlation with EPG was not significant. CD23, a 45-kDa transmembrane low-affinity IgE receptor expressed on the surface of naïve IgM+ IgD+ B cells (24), was found to be associated with the development of resistance to schistosomes (12). This phenomenon is mediated by its ability to elicit the synthesis and production of IgE (27). The CD23 receptors are also expressed on the surface of macrophages, platelets, monocytes, and eosinophils in response to schistosomiasis infection (37). Although not significant, sCD23 was higher among children with heavy infection. These findings corroborated the finding from a previous study in western Kenya, where CD23 was found to be elevated among schistosomiasis but did not correlate with egg burden (38).

The role of the assessed plasma biomarkers in immunity underpins the observed changes in their concentration during schistosomiasis. Particularly, the significant changes in sTREM levels suggest its potential use as a diagnostic marker of schistosome infection. Previous studies have demonstrated increased sTREM levels in sepsis and pneumonia patients (10, 39), albeit in non-schistosome conditions. Furthermore, our study showed consistency of sTREM concentration with egg burden (*p* < 0.0001). A strong positive correlation was also reported between sTREM and both eotaxin-1 and IL-6. These changes were consistent with the role of sTREM in triggering immunological response against infections by stimulating the secretion of IL-6 and eotaxin-1 (40). The increased secretion of sTREM in schistosome-infected children reaffirmed the applicability of sTREM in the diagnosis of schistosomiasis.

Changes in the concentration of FABP and LPS among schistosome-infected children were not significant, and their concentration did not correlate with infection intensity. Contrary to our findings, Aly et al. (41) reported a significant increase in FABP among *S. mansoni*-infected children. Nonetheless, mixed results have previously been reported on the association between LPS levels and schistosomal infection status. Onguru et al. (13) reported an increase in LPS among schistosome-infected individuals, while Klemperer et al. (28) showed that the level of LPS in schistosome-infected women did not change. We observed a subtle increase in the mean level of LPS among schistosome-infected children. There was no association between the biomarkers and hemoglobin levels, suggesting that they are not good indicators for anemia in schistosomiasis. However, Nakagawa et al. (42) reported a positive correlation between IL-6 and hemoglobin levels in individuals infected with schistosomiasis.

This study has provided evidence to suggest an increased concentration of sTREM in children infected with *S. mansoni* as well as its association with both eotaxin-1 and IL-6. These findings support the use of sTREM as a potential marker for the diagnosis of schistosomiasis. However, large-scale studies are required to validate the specificity of this candidate marker for the diagnosis of schistosomiasis infection. Future serological investigations of schistosomiasis could focus on comparing the concentration of these biomarkers in different *Schistosoma* species to establish possible cross-species variation.

## Conclusion

sTREM is a potential biomarker for diagnosis of schistosomiasis. A combination of biomarkers (sTREM and eotaxin-1) has the potential to complement the existing diagnostic methods.

## Data Availability Statement

The original contributions presented in the study are included in the article/**Supplementary Material**. Further inquiries can be directed to the corresponding author.

## Ethics Statement

The studies involving human participants were reviewed and approved by the Scientific and Ethics Review Unit (SERU) of the Kenya Medical Research Institute (KEMRI). Written informed consent to participate in this study was provided by the participants’ legal guardian/next of kin.

## Author Contributions

PM, MO, and LG-L participated in the study design. IO led the enrolment of subjects and the collection of specimens. BO, RH, and IO performed the laboratory assays of the specimens. BO and EM analyzed the data. BO, EM, IO, MO, PM, and LG-L wrote the manuscript. All authors participated in reviewing and editing the manuscript and concurred with the final manuscript.

## Funding

This study was supported by the National Institutes of Allergy and Infectious Diseases (R01AI116593) awarded to LG-L.

## Author Disclaimer

The findings and conclusions in this report are those of the authors and do not represent the views of the funding agency.

## Conflict of Interest

Authors RH and LG-L was employed by company Elegance Biotechnologies.

The remaining authors declare that the research was conducted in the absence of any commercial or financial relationships that could be construed as a potential conflict of interest.

## Publisher’s Note

All claims expressed in this article are solely those of the authors and do not necessarily represent those of their affiliated organizations, or those of the publisher, the editors and the reviewers. Any product that may be evaluated in this article, or claim that may be made by its manufacturer, is not guaranteed or endorsed by the publisher.
