# Supplementary material for: Potential Utility of Systemic Plasma Biomarkers for Evaluation of Pediatric Schistosomiasis in Western Kenya
Source: Front Immunol. 2022 May 6;13:887213. doi: 10.3389/fimmu.2022.887213 (PMC9121796; doi:10.3389/fimmu.2022.887213)
Supplement: Supplementary file 1 [file Table_1.docx]

**Supplementary Table 1:** Comparison of median plasma level of biomarkers between light intensity and uninfected children.

**Median level (25**

**th**

**-**

**75**

**th**

**Percentile)**

| **Biomarkers** | **Infected individuals (n =92)** | **Healthy controls (n =22)** | **p** |
| --- | --- | --- | --- |
| IL-6 | 0.66 (0.3 – 1.8) | 0.39 (0.0 – 4.8) | 0.6518 |
| sTREM | 10.00 (0 – 27.0) | 10.65 (0.0 – 73.4) | 0.9628 |
| Eotaxin-1 | 17.03(9.0 – 30.6) | 15.59 (10.8 – 23.3) | 0.5871 |
| FABP | 413.30 (290.9 – 535.5) | 419.06 (382.3 – 559.2) | 0.5867 |
| sCD23 | 2292.00 (1895.0 – 3138.0) | 2035.09 (1448.0 –  2939.0) | 0.1587 |
| LPS | 0.23 (0.0 – 0.5) | 0.18 (0.0 – 0.5) | 0.8994 |

The analysis was performed with Mann-Whitney U. *significant difference at p ≤ 0.05.
